# A novel specific aptamer targets cerebrovascular endothelial cells after ischemic stroke

**DOI:** 10.1038/s41598-023-36993-6

**Published:** 2023-06-20

**Authors:** Heng Hu, Silin Wu, Tae Jin Lee, Aaron M. Gusdon, Yuxin Liu, Huimahn A. Choi, Xuefang Sophie Ren

**Affiliations:** 1 Department of Neurosurgery, McGovern School of Medicine, University of Texas Health Science Center, MSB 7.134, 6431 Fannin St., Houston, TX 77030 USA; 2 Department of Computer Science and Electrical Engineering, West Virginia University, Morgantown, USA

**Keywords:** Stroke, Blood-brain barrier, Translational research, Nanoscience and technology

## Abstract

Cell specific-targeted therapy (CSTT) for acute ischemic stroke remains underdeveloped. Cerebrovascular endothelial cells (CECs) are key components of the blood–brain barrier and are the first brain cells affected by ischemic stroke. After stroke, CEC injury causes insufficient energy supply to neurons and leads to cytotoxic and vasogenic brain edema. Aptamers are short single-stranded RNA or DNA molecules that can bind to specific ligands for cell specific delivery. The expression of vascular cell adhesion molecule-1 (VCAM-1) is increased on CECs after stroke. Herein, we report that an RNA-based VCAM-1-aptamer can specifically target CECs in stroke brains following transient middle cerebral artery occlusion in mice. Our data demonstrate the potential of an RNA-based aptamer as an effective delivery platform to target CECs after stroke. We believe this method will allow for the development of CSTT for treatment of patients with stroke.

## Introduction

Ischemic stroke is a leading cause of death worldwide, causing significant morbidity and mortality. While significant progress has been made within intravenous and endovascular therapy aimed at revascularization after stroke, these treatments do not address infarct evolution and secondary cell damage and brain injury after stroke^1–3^. Cell specific-targeted therapy (CSTT) offers an attractive method for targeting the cell types most affected by stroke while avoiding off-target effects.

The blood–brain barrier (BBB) is composed of highly specialized CECs, which insulates brain tissue from circulating blood and prevents blood, bacteria, and toxins from reaching the central nervous system (CNS).​​ CECs are the first brain cells to be affected by stroke. CEC dysfunction following stroke contributes to insufficient energy supply to neurons and BBB disruption, which lead to cytotoxic and vasogenic brain edema^4^. Methods to specifically target CECs are currently lacking and may be an effective treatment strategy after stroke.

Aptamers are short single-stranded DNA or RNA molecules, which have excellent specificity and high binding affinity with proteins^5,6^. Affinity and specificity of aptamers are comparable to antibodies, with the advantage of enhanced pharmacokinetics due to their smaller size^5,6^. Therefore, aptamers are well-suited molecules for targeted drug delivery, diagnostic tools, and therapeutic purposes.

Vascular cell adhesion molecule-1 (VCAM-1; CD106) is a member of the immunoglobulin-like superfamily (IgSF) expressed on endothelial cells and regulates inflammation associated vascular adhesion as well as the transendothelial migration of leukocytes^3^. VCAM-1 is significantly elevated in the ischemic hemisphere at 6 h post-stroke and plays an important role in inflammation and progression of ischemic injury after acute stroke^3,7^; however, it has been shown that anti-VCAM-1 antibodies did not protect against ischemic damage in animal models of stroke^8^. Using systematic evolution of ligands by exponential enrichment technology, a previous study has screened for and discovered an RNA-based aptamer that has easy access to the N-terminal two-domain fragment of VCAM-1 with high specificity and nanomolar affinity in vitro^9^. However, the effect of this VCAM-1-aptamer has not been tested in vivo so far. The goal of this study is to test the ability of this RNA-based VCAM-1-aptamer to target CECs in vivo after stroke as well as evaluate effectiveness for treating ischemic stroke using a murine transient middle cerebral artery occlusion (tMCAO) model.

## Results

### VCAM-1-aptamer specifically targets cerebral vascular endothelial cells after stroke

**Figure 1:**
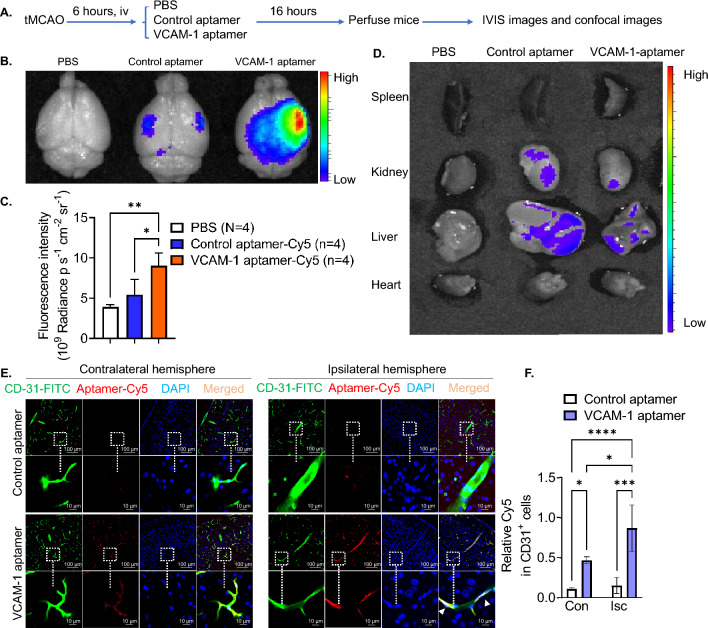
VCAM-1-aptamer specifically targets cerebral vascular endothelial cells after stroke. (**A**) Experimental design. (**B**) Representative fluorescence images in brains from stroke mice by IVIS imaging system at 22 h post-stroke. (**C**) Quantified fluorescence intensities of the brains from IVIS imaging. Data represent mean ± S.D.; n = 4 mice/group. ANOVA followed by post-hoc Tukey’s test. *P < 0.05; **P < 0.01. (**D**) IVIS images for peripheral organs from stroke mice. Fluorescence images in peripheral organs, spleen, kidney, liver and heart from stroke mice by IVIS imaging system at post-stroke 22 h. (**E**) Confocal immunofluorescent images of Cy5 colored in red and localization in CD31 positive CECs colored in green. Cell nuclei were stained with DAPI colored in blue. (**F**) The ratio of Cy5 MFI: CD31 MFI was calculated and the mean values of 20 views were reported for each mouse. Data represent the mean ± SD.

We performed one-hour tMCAO in mice and intravenously administered Cy5-labeled VCAM-1-aptamers, Cy5-labeled control aptamers, or PBS at 6 h post-stroke (Fig. 1A). The IVIS imaging system demonstrated that Cy5 fluorescence is enriched in ischemic hemispheres (Fig. 1B) and the mean fluorescence intensity (MFI) of Cy5 was significantly higher in mice treated with VCAM-1-aptamer compared with the PBS group and the control aptamer group in ischemic hemispheres (Fig. 1C). Peripheral organs (heart, liver, spleen, and kidney) did not show enriched Cy5 fluorescence (Fig. 1D). Confocal microscopy further confirmed that Cy5 fluorescence overlapped with CD31 + CECs in VCAM-1-aptamer treated stroke mice (Fig. 1E). Quantified relative expression of Cy5 in CD31^+^ CECs demonstrated that the MFI of Cy5 was significantly higher in stroke brains of VCAM-1-aptamer treated stroke mice (Fig. 1F). These data suggest that the VCAM-1-aptamer specifically targets CECs in stroke mice.

### VCAM-1-aptamer does not protect against stroke damage

**Figure 2:**
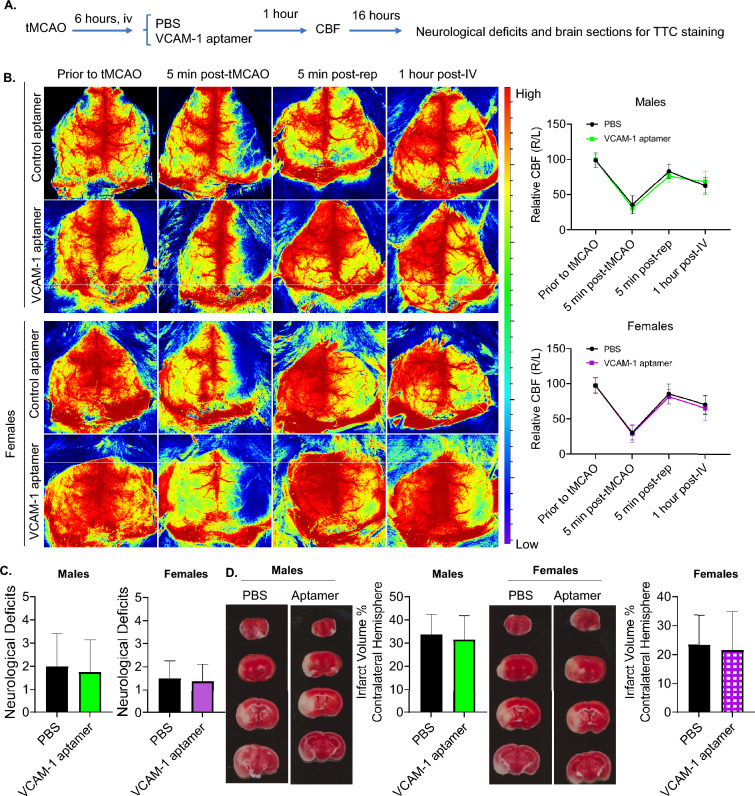
VCAM-1-aptamer does not affect cerebral blood flow and stroke outcomes in mice. (**A**) Experimental design. (**B**) Cerebral blood flow at four time points. Two-way ANOVA followed by post-hoc Sidak’s multiple comparisons test. Data are presented as mean ± SD, N = 8 per group. (**C**) Neurological deficits analyzed by Mann–Whitney U tests. N = 9 in males and N = 8 mice in females. (**D**) Brain infarct volume by TTC-staining. Student’s t-tests. Data presented as mean ± SD, N = 8 mice per group.

To test if this VCAM-1-aptamer affects stroke outcomes, we performed tMCAO in a separate cohort of mice and injected VCAM-1-aptamer (0.5 nmol) compared to PBS control (Fig. 2A). By analyzing cerebral blood flow (CBF) at four timepoints (prior to tMCAO, 5 min post-tMCAO, 5 min post-reperfusion, and 1-h post-intravenous administration) we found that VCAM-1-aptamer had no significant effect on CBF in both male and female mice (Fig. 2B). In male mice, one mouse from each group died prior to the 22-h experimental endpoint. Males and females had similar degrees of neurological deficits in both the PBS and VCAM-1-aptamer groups (Fig. 2C). Infarct volume did not differ in PBS and VCAM-1-aptmer treated groups when stratified by sex (Fig. 2D).

**Figure 3:**
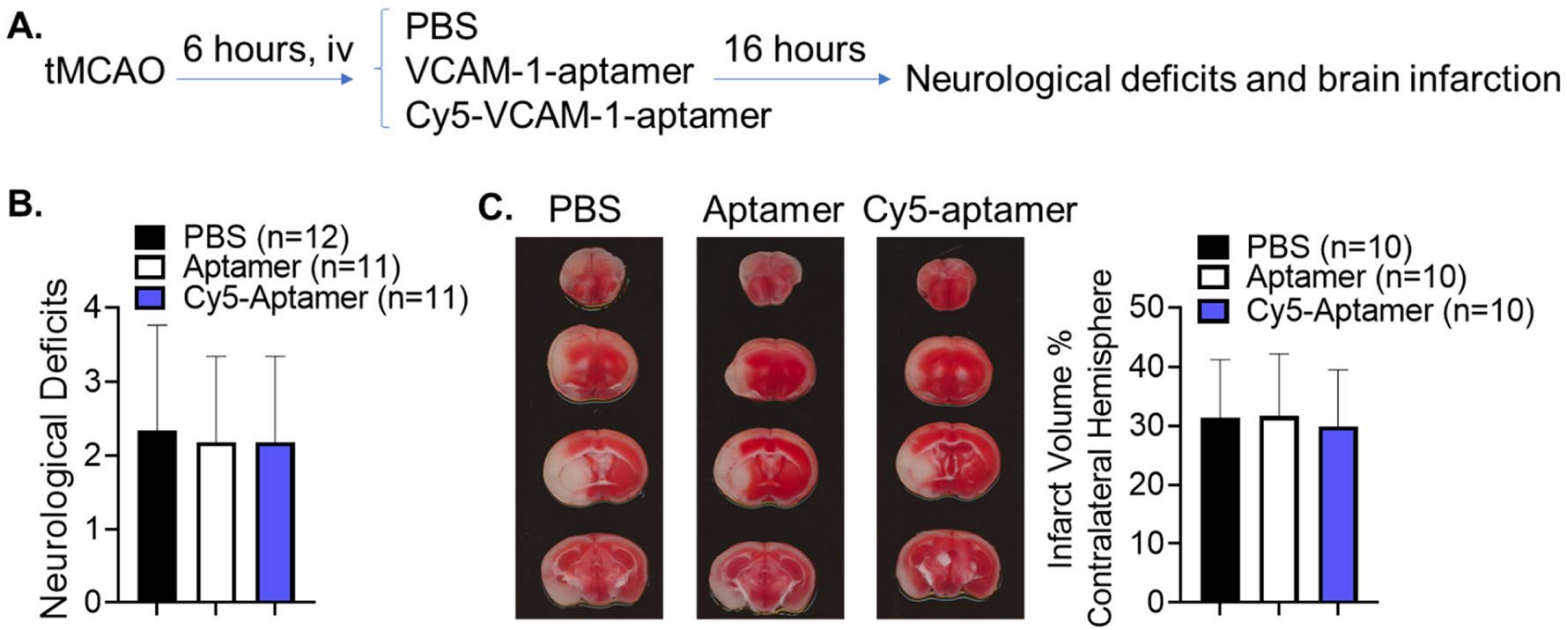
A high dose of VCAM-1-aptamer does not affect stroke outcomes in mice. (**A**) Experimental design. (**B**) Neurological deficits analyzed by Kruskal–Wallis test. Data presented as mean ± SD, N = 11–12 mice per group. (**C**) Brain infarct volume by TTC-staining. One way ANOVA followed by post-hoc Tukey’s tests. Data presented as mean ± SD, N = 10 mice per group.

We further evaluated if a higher dose of VCAM-1-aptamer affects stroke outcomes. We administered 2.5 nmol of VCAM-1-aptamer (with or without Cy5 label) to male stroke mice at 6 h post-stroke compared to PBS control (Fig. 3A). While two stroke mice died in PBS treated group and one stroke mouse from each aptamer treated group died prior to the 22-h endpoint, their neurological deficits were not significantly different among these three groups (Fig. 3B). Quantified infarct volumes did not significantly differ either (Fig. 3C). The data have also demonstrated that Cy5 conjugate does not impact neurological deficits (P > 0.99) and infarct volumes (P = 0.95) in stroke mice (Fig. 3B,C). Taken together, these data suggest that the VCAM-1-aptamer does not significantly affect mortality, functional deficits, or infarct volume in stroke mice.

## Discussion

Aptamers have several favorable features including high specificity and binding affinity with specific proteins, small size, low immunogenicity, relatively low cost for production, and ease of store^10^. Therefore, aptamers are one of the best candidates for targeting the specific ligands or receptors that have been applied to therapeutic, biosensing, and engineering fields^10^. The selected VCAM-1-aptamer has high affinity for N-terminal two-domain fragment of VCAM-1, suggesting a potential inhibitory activity on their targets, either by directly blocking VCAM-1 or indirectly blocking its ligand α4β1 integrin expressed on leukocytes to bind VCAM-1 on CECs. While our results have shown high specificity of this VCAM-1-aptamer with CECs in ischemic hemispheres (Fig. 1), we unexpectedly did not observe a significant impact of this aptamer (with or without Cy5 conjugate) on stroke outcomes (Figs. 2, 3), which represents a barrier to developing this aptamer into therapeutic applications. However, our data demonstrated it has low toxicity in stroke mice, suggesting that this aptamer could be a useful and safe tool to target CECs following ischemic stroke.

It was previously reported that VCAM-1 has negligible expression evident in normal brain tissue^11^, but is markedly elevated in CECs after stroke and involved in inflammatory and immune responses^3^, which are implicated in the evolution of infarction. Given the high specificity of the VCAM-1-aptamer to CECs shown in our study, it could be utilized as a tool to gauge the degree of inflammation and tissue damage, possibly reflecting the severity of stroke. In vivo imaging of VCAM-1 expression using molecular imaging techniques such as positron emission tomography (PET) or magnetic resonance imaging (MRI) may, therefore, allow for the non-invasive diagnosis and monitoring of stroke and inflammatory diseases. Previous studies have investigated antibodies against VCAM-1. Aniti-VCAM-1 antibody conjugated with a contrast agent has been used as a molecular imaging tool to monitor vascular inflammation in stroke rats^7^. Despite the promising results of anti-VCAM-1 antibody conjugates as molecular imaging tools, the risk of immunogenicity has posed a challenge in the field. Our study has demonstrated herein that the VCAM-1-aptamer does not influence cerebral blood flow, neurological deficits, and infarct volume in the murine tMCAO model. There was no dose response effect on infarct volume when mice received 0.5 nmol (Fig. 2) compared with 2.5 nmol of aptamer (Fig. 3) (P = 0.68). The VCAM-1-aptamer conjugated to Cy5 fluorescence did not significantly impact stroke outcomes either (Fig. 3). It may therefore offer a better option for conjugation to a biosensor for developing molecular MRI, PET, or other in vivo imaging systems.

The extracellular domain of VCAM-1 contains seven Ig-like domains^12^. Ig-like domain pairs of 1 and 4, 2 and 5, and 3 and 6 are highly homologous with each other^12^. Domains 1 and 4 of VCAM-1 are involved in the direct binding of α4β1 integrin, resulting in leukocyte adhesion^12,13^. The VCAM-1-aptamer used in this study is very small (39 oligonucleotides) and only corresponds to VCAM-1 domains 1 and 2, which might not be able to block binding α4β1 integrin and reduce leukocyte adhesion. This could explain why the aptamer failed to protect against stroke damage (Figs. 2, 3) while showing high affinity for CECs (Fig. 1) in our stroke model. Conjugating the VCAM-1-aptamer with other proteins and peptides could potentially block VCAM-1 from binding to α4β1 integrin, offering new strategy to develop novel therapeutics. It remains to be verified if aptamers corresponding to other domains of VCAM-1 could offer therapeutic effects for stroke.

The expression of VCAM-1 on CECs is elevated not only in neurological disorders including stroke, subarachnoid hemorrhage, brain tumor, multiple sclerosis, and traumatic brain injury^14,15^ but also involved in various immunological disorders, such as rheumatoid arthritis, asthma, transplant rejection, etc.^16^. Our study warrants further investigation and development as in vivo targeting platform for CECs, and we believe that the VCAM-1-aptamer may be applied to numerous applications in the future.

## Methods

### Guidelines

All animal experiments were approved by the Animal Care and Use Committee at the University of Texas Health Science Center at Houston (accredited by the American Association for Accreditation of Laboratory Care) and performed in accordance with the National Institutes of Health Guide for the Care and Use of Laboratory Animals. Both male and female C57/BL6J mice (~ 3 to 6 months old) were used in this study. The ARRIVE guidelines (Animal Research: Reporting of In Vivo Experiments) were followed to report animal experiments.

### Transient middle cerebral artery occlusion (tMCAO)

Focal cerebral ischemia was induced under isofluorane anesthesia with standard operation procedures. The mice were subjected to 1 h transient middle cerebral artery occlusion (tMCAO) using silicon coated sutures (Cat. #702334, diameter 0.23 mm, Doccol Corporation, MA) followed by reperfusion for 22 h as published previously^1,4,17^. Body temperature was controlled at 37 ± 0.5 °C during occlusion. Experimenters were blinded for data analysis. The inclusion/exclusion criteria were followed as published previously^1,4,17^. Bupivacaine (2 mg/kg, s.c.) was administered to relieve pain after surgery.

### Cerebral blood flow

Cerebral blood flow was recorded by a Laser Speckle Imager (RWD Life Science Co, San Diego CA) at 4 time points: prior to tMCAO, 5 min after occlusion, 5 min after reperfusion, and 1 h after aptamer administration (7 h after stroke).

### Aptamer administration in vivo

VCAM-1-aptamer (sequence^9^: 5′-AGGGAAUCUUGCCUAGGGAGGGAGUAGCGAAAGGGCUCA-3′) and scramble control (5′-GGAGACGCGUGCCAUGGUAAGGAAGUGCGAGAAGUACGU-3′) were synthesized from Horizon Discovery Biosciences Limited (Cambridge, UK) with in vivo HPLC-grade purification. 2′-Fluoro-U and 2′-Fluoro-C were used to increase the stability of aptamers. We followed the MAPS guidlines^18^ to prepare the aptamers. Aptamers were labeled with Cy5 (Figs. 1, 3) or without Cy5 (Figs. 2, 3), dissolved in 100 µL of PBS, and administered at 6 h post-stroke via tail vein injection. In Figs. 1 and 2, 0.5 nmol of aptamers were given. In Fig. 3, 2.5 nmol of aptamers were administered.

### IVIS imaging

Mice were perfused with 10 mL of PBS under deep anesthesia. Brains, livers, kidneys, spleens, and hearts were collected and measured immediately on an IVIS Spectrum Imaging System (PerkinElmer, Austin TX). An excitation wavelength of 640 nm and emission wavelength of 680 nm were chosen in acquiring signals. The total radiant efficiency (photon/s/cm^2^/sr/μW/cm^2^) of samples was determined using Living Image software (PerkinElmer, Cleveland, USA).

### Immunohistochemistry staining

Brains were fixed in 10% paraformaldehyde for 2 days then in 30% sucrose for 2 days. Brain sections (20 μm) were obtained on Leica cryostat (− 20 °C) then blocked with 5% goat serum staining buffer and stained with anti-mouse-CD31 (R&D systems, Minnesota, USA) overnight, then washed with PBS and stained with FITC-conjugated goat-anti-rabbit (Jackson Immunoresearch, Inc. Pennsylvania, USA) for 2 h. Brain sections were washed with PBS and mounted on glass slides using prolong gold anti-fade reagent (Life technologies, California, USA). The slides were photographed with a Nikon A1Rsi Confocal microscope (Nikon, Japan) using the Nis Elements software.

### Neurological deficits

Neurological deficits were determined at 22 h post-tMCAO according to a 0- to 5-point scale neurological score system as published^1,4,17^. The experimenters were blinded to treatments.

### Brain infarct volume

The brain sections were stained with 2% Tetranitroblue tetrazolium chloride (TTC, Cat. #T4000, Sigma, Saint Louis, MO) at 37 °C for 30 min then photographed by an image scanner (CanoScan 9000F, Japan). Infarct volume was analyzed using ImageJ (National Institutes of Health) in a double-blinded manner. The infarct volume was expressed as a percentage of contralateral hemisphere.

### Statistical analysis

We performed statistical analysis using Prism 5 software (Graphpad software, California, USA). Differences between two groups were analyzed using unpaired Student’s t-tests or Mann–Whitney U tests, one-way analysis of variance (ANOVA), or two-way ANOVA as indicated in the figure legends. P < 0.05 was statistically significant.

## Data Availability

The datasets used and/or analyzed during the current study are available from the corresponding author on reasonable request.
